# Role of Stromal Cells in Determining Tumor and Cancer Stem Cell Behaviors and Therapeutic Response

**DOI:** 10.3390/cancers12113162

**Published:** 2020-10-28

**Authors:** Stephan J. Reshkin, Rosa Angela Cardone

**Affiliations:** Department of Biosciences, Biotechnology and Biopharmaceutics, University of Bari, Via E. Orabona 4, 70126 Bari, Italy

While research previously focused extensively on the tumor cells, over the last two decades, the tumor microenvironment (TME) has received increasing attention with a particular emphasis in its role in tumor development, metabolism, progression, and treatment response [1,2,3,4,5]. The TME is composed of stromal extracellular matrix (ECM) components (i.e., laminins, fibronectin, collagens, proteoglycans, and elastin) and of stromal cells (fibroblasts, endothelial cells, macrophages and lymphocytes). Most of these cellular components produce tumor-supportive ECM and secrete growth factors and chemokines that further alter the ECM and generate oncogenic signals, thus playing key roles in tumor transformation, cell proliferation and tissue invasion [1,2,3,4,5]. The ECM itself provides structural support, stability, flexibility and shape for the tissue and, by doing this, also mediates cell polarity, intracellular signaling and cell migration/invasion [6]. This biophysical interplay between the ECM and the cells establishes a dynamic reciprocity as its maintenance and homeostasis involve a tight balance between the ECM protein biosynthesis, 3D organization, cross-linking, and degradation [6]. The tumor ECM is altered during malignant progression, and its interaction with the cancer cells plays an essential role in tumor metabolism, development, progression, recruitment and metabolic reprograming of tumor and stromal cells and treatment response [1,2,3,4,5,6]. 

The TME also includes the tumor metabolic microenvironment (TMM), which is characterized by dynamic, interacting areas of hypoxia, low extracellular pH (pHe) and low nutrients. This adverse pathophysiological TMM is formed by the vascular abnormalities, inadequate microcirculation, high vascular permeability and increased interstitial fluid pressure [7]. Furthermore, this TMM leads to upregulation of glycolytic capacity (Warburg effect) and lactate accumulation and energy depletion [7]. Of these conditions, the best characterized is hypoxia, which contributes, among other effects, to mutagenesis, suppression of apoptosis, epithelial-to-mesenchymal transition and the selection of tumor-promoting and chemoresistant clones, the cancer stem cell (CSC) [8,9]. CSCs appear both early and late during tumor development and progression and undergo reversible and dynamic phenotypic transitions to adapt and proactively model their original tumor niches in tumor-supportive microenvironments as the disease progresses [10,11]. In these altered tumor-niches, CSCs, due to their intrinsic ability to self-renew and differentiate into a wide variety of cell types and their extrinsic interactions with the changing extracellular conditions, give rise to the tumor heterogeneity, plasticity and malignancy typical of many types of solid tumors [12]. Tumor stemness is also supported by the extracellular acidosis of the TME, which is now considered a new hallmark of solid tumors [13,14]. The highly acidic conditions (pHe 6.6–6.8) of the TME result from the elevated metabolic rates in the highly proliferative cancer cells, in conjunction with often greatly increased rates of net cellular acid extrusion [15,16,17]. Studies in various cancers have suggested that while the acid extrusion mechanisms employed are generally the same as those in healthy cells, the main transporters upregulated vary with the cancer type. The main transporters include the following: Na^+^/H^+^ exchangers (NHE1), various HCO_3_^−^ transporters, H^+^ pumps, and lactate-H^+^ cotransporters. The mechanisms leading to their dysregulation in cancer (changes in transporter expression levels, trafficking and membrane localization, and/or posttranslational modifications) are still poorly understood [15,16,17]. Accumulating evidence has revealed that in addition to supporting their elevated metabolic rate, the increased acid efflux endows the cancer cells with enhanced invasive capacity, proliferation and chemotherapy resistance [5]. Indeed, the acidic tumor microenvironment is an important contributing factor to metastasis of cancer cells via a combination of toxicity to adjacent normal cells, degradation of the ECM through induced secretion and activation of proteases [18,19], increased cancer cell motility and invasion, reduced immunological defenses [3,7,20] and stromal cell activation and/or recruitment to drive malignant progression [16]. In line with this, Hulikova et al. [21] demonstrated that stromal myofibroblasts can act as proton reservoirs to affect extracellular acidification and transmit protons across the stroma. Interestingly, cancer-adjacent normal tissue that is subjected to the increased acidity is more susceptible to cancer invasion [20]. Indeed, cancer cells in the invasive edge of tumors generally have higher upregulation of proteins in acid-generating pathways such as the glucose transporter GLUT-1 and the pH-secreting proteins. Moreover, the effectiveness of certain chemotherapeutic agents is negatively impacted by the tumor extracellular acidosis via (i) a reduced efficacy of common weak base chemotherapies; (ii) the accumulation of the charged drugs in the extracellular space; and (iii) an increased efflux of known drug transporters such as p-glycoproteins, which confers an additional mechanism of drug resistance [6]. The reciprocal interaction(s) of stromal cell types, the ECM, the metabolic microenvironment and neoplastic cells (parenchymal and CSCs) is highly complex and most likely varies between tumor types. The challenge therefore is to identify the most important and general aspects of these interactions in the tumor microenvironment and how they co-evolve during metastatic progression.

Given the highly complex nature of the tumor microenvironment and its components, it is likely that many microenvironmental factors can contribute to cancer growth and progression and that they interact and co-evolve in very complex ways. Mapping the real-time spatial changes in intratumoral pH, pO_2_, and glucose concentrations will improve our understanding of the heterogeneity of tumor properties and behaviors and their roles in progression and therapeutic resistance. This will give us a mechanistic understanding of the forces shaping the TMM landscape during tumor progression and how the various complex components of the tumor microenvironment co-evolve to drive progression and metastasis and their role in resistance to traditional therapies. This understanding should help in developing novel reagents and strategies for diagnosis and more effective therapies, exploiting the components of the TME as a co-target with conventional chemotherapies for controlling tumor progression and overcoming therapeutic resistance.
